# PopB-PcrV Interactions Are Essential for Pore Formation in the Pseudomonas aeruginosa Type III Secretion System Translocon

**DOI:** 10.1128/mbio.02381-22

**Published:** 2022-09-26

**Authors:** Emma Kundracik, Josephine Trichka, José Díaz Aponte, Alicia Roistacher, Arne Rietsch

**Affiliations:** a Department of Molecular Biology and Microbiology, Case Western Reserve University, Cleveland, Ohio, USA; b Experimental Immunology Branch, National Cancer Institute, National Institutes of Health, Bethesda, Maryland, USA; c Department of Pathology, Case Western Reserve University, Cleveland, Ohio, USA; d Department of Physiology and Biophysics, Case Western Reserve University, Cleveland, Ohio, USA; Emory University School of Medicine

**Keywords:** T3SS, translocon, pore formation

## Abstract

The type III secretion system (T3SS) is a syringe-like virulence factor that delivers bacterial proteins directly into the cytoplasm of host cells. An essential component of the system is the translocon, which creates a pore in the host cell membrane through which proteins are injected. In Pseudomonas aeruginosa, the translocation pore is formed by proteins PopB and PopD and attaches to the T3SS needle via the needle tip protein PcrV. The structure and stoichiometry of the multimeric pore are unknown. We took a genetic approach to map contact points within the system by taking advantage of the fact that the translocator proteins of P. aeruginosa and the related Aeromonas hydrophila T3SS are incompatible and cannot be freely exchanged. We created chimeric versions of P. aeruginosa PopB and A. hydrophila AopB to intentionally disrupt and restore protein-protein interactions. We identified a chimeric B-translocator that specifically disrupts an interaction with the needle tip protein. This disruption did not affect membrane insertion of the B-translocator but did prevent formation of the translocation pore, arguing that the needle tip protein drives the formation of the translocation pore.

## INTRODUCTION

The syringe-like type III secretion system (T3SS) is used by Pseudomonas aeruginosa to inject host cells with effector proteins—ExoS, ExoT, ExoU, and ExoY—that weaken epithelial barriers and hinder immune clearance by neutrophils and macrophages (1). This system is a prime target for antivirulence drug development (2–4). Particularly attractive as a target, the translocon creates a pore in the host cell membrane and is partially accessible from the extracellular space. However, the translocon is poorly understood (5–7). Complex processes, such as the needle tip-mediated insertion of translocator proteins into the host cell membrane or triggering of effector secretion, cannot be recapitulated *in vitro*. The structure of the translocation pore remains elusive. The intact translocation pore has yet to be purified. The Salmonella translocon was visualized in the context of an infected cell by cryo-electron tomography, but the structure lacked molecular resolution (8). A further complication, in the case of P. aeruginosa, is that the copy number of the assembled system is low, hampering direc*t in situ* imaging studies (9, 10).

**FIG 1:**
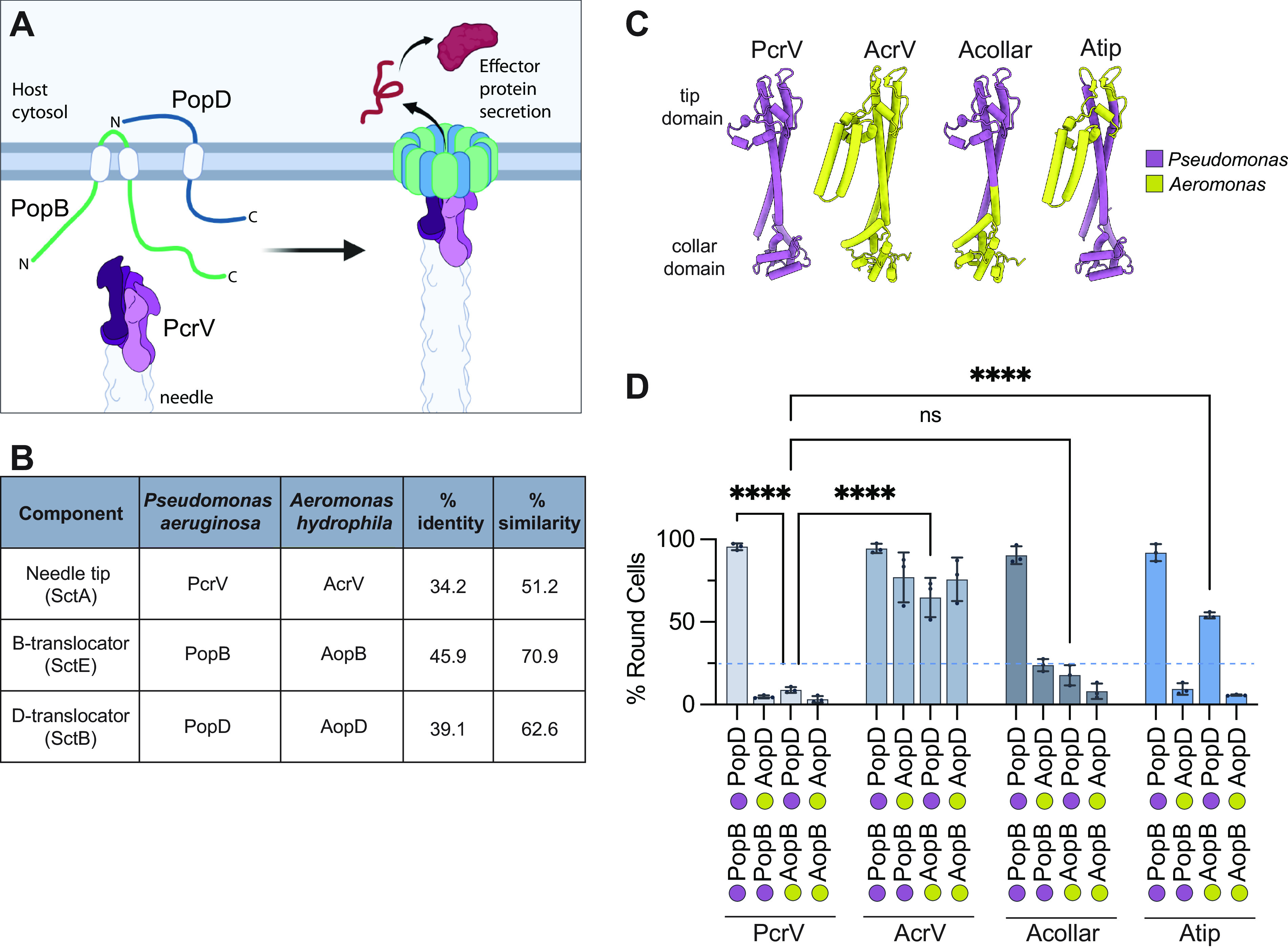
Translocator proteins from P. aeruginosa and A. hydrophila are not interchangeable. (A) Model of component parts of the translocon (left) and assembled translocation pore (right). PopB is inserted in the membrane with N- and C-termini toward the extracellular space (54). PopD has its C terminus in the extracellular space (25). The pentameric arrangement of PcrV is based on alignment to the Salmonella needle tip structure (55). (B) Amino acid sequence identity and similarity for P. aeruginosa (accession number AE004091) and A. hydrophila AH-3 (accession number AY528667) translocators. Sequences were aligned with ClustalW. (C) AlphaFold2 models of the needle tip protein from P. aeruginosa (PcrV), A. hydrophila strain AH-3 (AcrV), and two chimeric-fusion V proteins. The hybrid Acollar protein has an AcrV substitution in the N-terminal globular domain [residues 1 to F143 from AcrV are fused to PcrV(Q124–end)]. The hybrid Atip contains the bulkier tip domain of AcrV substituted for the tip domain of PcrV (residues 1 to A158 from PcrV, A179 to S310 from AcrV, and V244 to the end from PcrV). Monomer models were created from protein sequences using AlphaFold2 implemented in Google Colab Notebooks (33, 56) and visualized with UCSF ChimeraX (57). The position of the collar domain is uncertain and varies across models. The tip domain has a high degree of confidence and is static between models (55). Pseudomonas segments are shown in purple, and Aeromonas segments in yellow. (D) All strains lack the pore-forming translocator operon (*ΔpcrH-popBD*) and produce the indicated needle tip protein: PcrV (strain RP3624), AcrV (strain RP7360), Acollar (strain RP6166), or Atip (strain RP6425). These background strains were complemented with plasmids carrying the indicated translocator proteins along with both export chaperone homologs, *pcrH* and *acrH* (Table S1). A549 epithelial cells were infected, and cellular rounding (indicating delivery of ExoS) was monitored by microscopy after 2 h. In our analysis, we consider a translocator system nonfunctional if it leads to less than 25% cell rounding at 2 h (dotted line). Statistical significance was calculated by two-way analysis of variance (ANOVA) with the Tukey multiple-comparison test. *n* = 3 biological replicates. Error bars show standard deviations. ****, *P* < 0.0001; ns, not statistically significant (*P* > 0.05). Statistical data for all comparisons are listed in Table S3. Schematic figures were created using BioRender.com.

The formation of the translocation pore has been studied *in vitro* using purified proteins (11–14). In the case of P. aeruginosa, two transmembrane proteins, PopB and PopD, oligomerize to form a pore in the host cell membrane (Fig. 1A). PopB and PopD can also form pores in lipid bilayers (15–17), under the right conditions, and these pores require both translocator proteins, just as both PopB and PopD are required for translocation in the context of host cells. The stoichiometry of translocator proteins in *in vitro*-formed pores was estimated using photobleaching, and the results suggest that these pores consist of 8 copies of PopB and 8 copies of PopD (18). Genetic studies have also shed light on the composition and function of the translocon. The needle tip protein is required for translocator insertion in every T3SS examined to date (19–24). Indeed, the P. aeruginosa needle tip protein PcrV connects the translocation pore to the T3SS needle and is needed for the insertion of PopB and PopD into the host cell membrane. Recent data suggest that the insertion of PopD is further facilitated by PopB (15). The needle tip protein plays a role in sensing host cell contact, so that effector proteins are unleashed only after the translocon is fully assembled (25–27). Triggering effector secretion requires a conformational change in the translocation pore that, in the case of Pseudomonas, is transmitted via the C-terminus of PopD to the collar domain of PcrV (25). Conformational changes likely also trigger effector secretion in other species. This has been best characterized in Shigella, where intermediate filament binding and actin polymerization mediate translocon stability and the opening of the translocation pore through distinct conformational changes in the translocon (28, 29). These systems differ in the nature of the host factors required to trigger effector secretion, however, since interfering with actin polymerization does not inhibit effector translocation in Pseudomonas (25, 30). The nature of the host cell process that triggers effector secretion in the P. aeruginosa system remains enigmatic.

10.1128/mbio.02381-22.8 TABLE S1 List of bacterial strains and plasmids used in the study. Download Table S1, PDF file, 0.05 MB. Copyright © 2022 Kundracik et al. 2022 Kundracik et al. https://creativecommons.org/licenses/by/4.0/ This content is distributed under the terms of the Creative Commons Attribution 4.0 International license.

10.1128/mbio.02381-22.10 TABLE S3 All pairwise comparisons for translocator exchange cytotoxicity testing presented in Fig. 1D. Download Table S3, XLSX file, 0.01 MB. Copyright © 2022 Kundracik et al. 2022 Kundracik et al. https://creativecommons.org/licenses/by/4.0/ This content is distributed under the terms of the Creative Commons Attribution 4.0 International license.

In this study, we took a genetic approach to map interactions between translocon proteins. We exploited the incompatibilities between the homologous T3SS systems of P. aeruginosa and Aeromonas hydrophila to map an interaction between the needle tip protein and two small regions of PopB. Functional assays of our genetic hybrids revealed that the PopB-PcrV interaction is crucial for the formation of the translocation pore, but not for insertion of the translocators into the host membrane. We therefore propose for the first time that the needle tip protein is actively involved in assembling the translocation pore.

## RESULTS

### P. aeruginosa and A. hydrophila translocator proteins are incompatible.

P. aeruginosa translocator proteins are not interchangeable with their Yersinia homologs (21, 25, 31). Previously, we used this observation to genetically map the protein-protein contacts that were disrupted by combining Pseudomonas and Yersinia translocators. This led to the discovery of several new protein-protein interactions, two of which—an interaction between PopD and PcrV and a PopD homodimer interaction—are important for sensing host cell contact. Here, we extend this analysis by examining the incompatibility between P. aeruginosa and A. hydrophila translocator homologs (Fig. 1B and Fig. S1 and S2 in the supplemental material). Compared to PcrV, AcrV has a 49-residue insertion in the hypervariable portion of the tip domain (32). This segment is predicted by AlphaFold2 to fold into an alpha-helical hairpin (Fig. 1C) (33). The rest of the tip domain and central helices are predicted to fold similarly in PcrV and AcrV.

**FIG 2:**
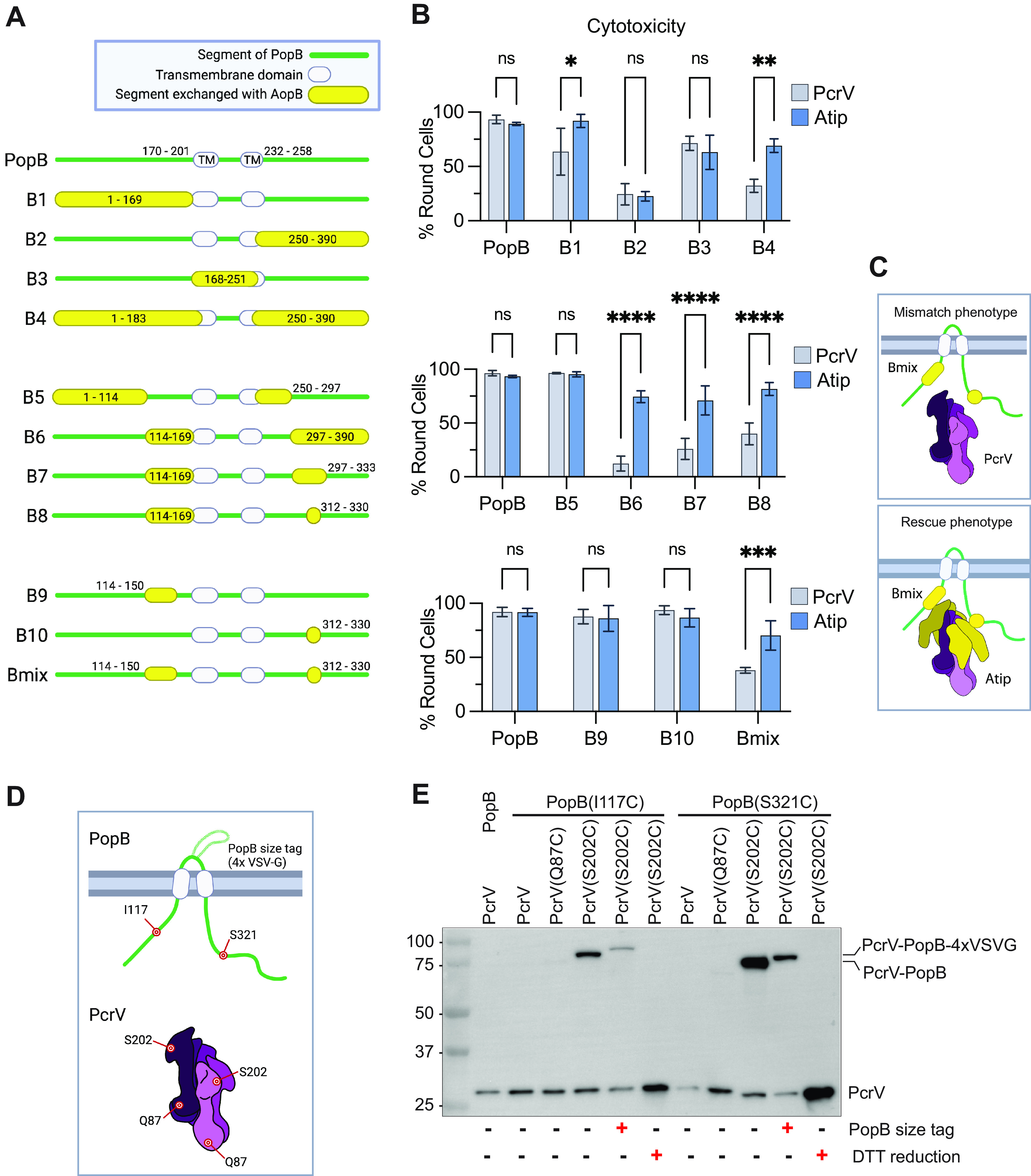
The AopB-Atip interaction is mapped to two small regions of the B-translocator. (A) Schematic diagram of PopB-AopB chimeras. Residues are numbered according to PopB amino acid sequence. PopB transmembrane domains are predicted to be residues 170 to 201 (TM1) and residues 232 to 258 (TM2) (58). Green lines indicate PopB. Yellow bubbles indicate AopB. Transmembrane domains are indicated by white bubbles. (B) Background strains PAO1F Δ*exsE* Δ*exoT exoY* Δ*pcrH-popBD* with wild-type *pcrV* (strain RP3624) or *Atip* (strain RP6425) were complemented with *acrH*, *pcrH*, and *popD* and the indicated *popB-aopB* chimera. A549 cells were infected for 2 h, and the proportion of round cells assessed. *n* = 3 biological replicates. Error bars show standard deviations. Statistical significance was calculated using two-way ANOVA with the Sidak multiple-comparison test. Significance thresholds: *, *P* < 0.05; **, *P* < 0.005; ***, *P* < 0.0005; ****, *P* < 0.0001; ns, not statistically significant (*P* > 0.05). (C) Schematic diagram of Bmix chimera with wild-type PcrV or chimeric Atip, illustrating the tip-dependent phenotype. The Bmix/PcrV combination has reduced cytotoxicity, presumably due to incompatibilities introduced by the homologous substitutions (mismatch phenotype). The Bmix/Atip combination performs significantly better in the cytotoxicity assay (rescue phenotype). (D) Cysteine mutations were introduced into PopB and PcrV in the locations shown schematically. In subsequent experiments, PcrV proteins having a single cysteine mutation (Q87C or S202C) were paired with PopB mutants having a single cysteine mutation (I117C or S321C). A variant of PopB includes a 5.3-kDa size tag consisting of four copies of the vesicular stomatitis virus glycoprotein (4×VSV-G) inserted after residue 225 (25). (E) Background strains PAO1F Δ*exsE exoS*(G/A−) Δ*exoT exoY* Δ*pcrH-popBD* with wild-type *pcrV* (strain RP3670), *pcrV*(*Q87C*) (strain RP12661), or *pcrV*(*S202C*) (strain RP12598) were complemented with *acrH*, *pcrH*, and *popD* and the indicated version of *popB*. A549 cells were infected in the presence of copper for 2 h. After infection, off-target disulfide bond formation was blocked by the addition of iodoacetamide. Membrane extract samples were prepared in SDS sample buffer lacking or containing dithiothreitol (DTT) as indicated. The blot is representative of three independent experiments. Molecular-weight size markers (in kilodaltons) are indicated. “*exoS*(G/A−)” denotes the mutant *exoS*(*R146K/E379D/E381D*), which is enzymatically inactive (30). Schematic figures were created using BioRender.com.

10.1128/mbio.02381-22.1 FIG S1 Protein sequence alignment for PcrV-AcrV. Related to Fig. 1 and 2. PcrV (GenBank accession no. AE004091) and AcrV (GenBank accession no. AY528667) were aligned using ClustalW. The amino acid sequences are 36% identical (50% similar). The construct Atip is indicated by purple (segments from PcrV) and yellow (segments from AcrV). Download FIG S1, TIF file, 0.9 MB. Copyright © 2022 Kundracik et al. 2022 Kundracik et al. https://creativecommons.org/licenses/by/4.0/ This content is distributed under the terms of the Creative Commons Attribution 4.0 International license.

10.1128/mbio.02381-22.2 FIG S2 Protein sequence alignment for PopB-AopB. Related to Fig. 1 and 2. PopB (GenBank accession no. AE004091) and AopB (GenBank accession no. AY528667) were aligned using ClustalW. The amino acid sequences are 46% identical (71% similar). The construct Bmix is indicated by green (segments from PopB) and yellow (segments from AopB). Boxes indicate predicted transmembrane domains from the data with UniProt accession numbers Q9I324 (PopB) and Q699Q8 (AopB). Download FIG S2, TIF file, 1.1 MB. Copyright © 2022 Kundracik et al. 2022 Kundracik et al. https://creativecommons.org/licenses/by/4.0/ This content is distributed under the terms of the Creative Commons Attribution 4.0 International license.

To determine whether translocator proteins from A. hydrophila can substitute for their respective P. aeruginosa homologs, we constructed P. aeruginosa strains carrying various combinations of the translocator proteins and needle tips, including PcrV-AcrV chimeras designated “Atip” and “Acollar.” These strains were assayed for their ability to intoxicate epithelial cells. Compatible sets of proteins induce cell rounding by mediating the delivery of ExoS, which induces actin depolymerization. Incompatible systems induce cell rounding more slowly or not at all (Fig. S3).

10.1128/mbio.02381-22.3 FIG S3 Additional controls and kinetics for cytotoxicity assay. Related to Fig. 1. (A) The cytotoxicity experiment was performed as described in the legend to Fig. 1 using background strains RP3624 (PcrV) and RP6425 (Atip). The wild-type strain is RP2318. The Δ*pcrV* strain is RP3223. The Δ*popB* and Δ*popD* strains have the background strain RP3624 complemented with a plasmid carrying either *popB* or *popD* along with both export chaperone homologs, *pcrH* and *acrH*. *n* = 3 biological replicates. Error bars show standard deviations. (B and C) Kinetics of cell rounding in response to P. aeruginosa infection. The cytotoxicity experiment was performed as described in the legend to Fig. 1 but was stopped at various time points up to 3 h. HBD denotes *pcrH-popB-popD*. *n* = 4 biological replicates. Error bars show standard deviations. The data set is split into two graphs for clarity. Download FIG S3, TIF file, 0.6 MB. Copyright © 2022 Kundracik et al. 2022 Kundracik et al. https://creativecommons.org/licenses/by/4.0/ This content is distributed under the terms of the Creative Commons Attribution 4.0 International license.

The native Pseudomonas translocators (PopB and PopD) are compatible with both PcrV and AcrV, as well as both hybrid needle tip proteins (Fig. 1D). In contrast, Aeromonas AopB and AopD each display specific incompatibilities. AopD is only compatible with AcrV. That is, the two systems containing both AopD and AcrV induce ~75% cell rounding within 2 h, whereas the strain producing AopD and PcrV is nonfunctional. Interestingly, AopD function is not fully restored by either hybrid needle tip, arguing that AopD disrupts interactions with both the collar and tip domains of PcrV. AopB is similarly incompatible with PcrV but functional when produced in the context of its cognate needle tip protein, AcrV. Notably, AopB is incompatible with PcrV and Acollar but significantly rescued by AcrV or Atip. This finding argues for an interaction between the B-translocator and the tip domain of the needle tip protein. We decided to pursue this interaction further, since no function has been assigned to the tip domain of the needle tip protein for any T3SS.

### The tip domain of the needle tip protein interacts with PopB.

We next sought to narrow down the specific region of AopB that clashes with the PcrV tip domain. We used an iterative approach to create a panel of PopB-AopB chimeric proteins, which were assayed in the context of the wild-type PcrV and the chimeric needle tip protein Atip (Fig. 2A and B and Fig. S4 and S5). Notably, this approach allowed us to exclude proteins that are likely misfolded, degraded, or no longer secreted, since these chimeras cannot be complemented through the addition of the permissive Atip needle tip protein.

10.1128/mbio.02381-22.4 FIG S4 Refining the region of interest for the PopB-AopB interaction. Related to Fig. 2. (A) Schematic diagrams of PopB-AopB chimeras. This set of chimeras was designed to refine the boundaries of the PopB-PcrV interaction, focusing on the N-terminal substitution. Created with BioRender.com. (B) Cytotoxicity data for PopB-AopB chimeras. The experiment was performed as described in the legend to Fig. 1 using background strains RP3624 (PcrV) and RP6425 (Atip). *n* = 3 biological replicates. Error bars show standard deviations. Statistical differences were analyzed with two-way ANOVA and the Sidak multiple-comparison test: *, *P* < 0.05; **, *P* < 0.005; ***, *P* < 0.0005; ****, *P* < 0.0001; ns, not statistically significant (*P* > 0.05). Download FIG S4, TIF file, 0.7 MB. Copyright © 2022 Kundracik et al. 2022 Kundracik et al. https://creativecommons.org/licenses/by/4.0/ This content is distributed under the terms of the Creative Commons Attribution 4.0 International license.

10.1128/mbio.02381-22.5 FIG S5 Evidence for a PopB-PopB interaction. Related to Fig. 2. (A) Schematic diagrams of PopB-AopB chimeras. (B) Cytotoxicity data for PopB-AopB chimeras. The experiment was performed as described in the legend to Fig. 1 using background strains RP3624 (PcrV) and RP6425 (Atip). *n* = 3 biological replicates. Error bars show standard deviations. Statistical differences were analyzed with two-way ANOVA and the Sidak multiple-comparison test: *, *P* < 0.05; **, *P* < 0.005; ***, *P* < 0.0005; ***, *P* < 0.0001; ns, not statistically significant (*P* > 0.05). (C) Schematic diagram showing two possible PopB-PopB interactions. In the first scenario (left), two adjacent monomers of PopB interact with each other. In the second scenario (right), PopB folds on itself and has an intramolecular interaction. Created with BioRender.com. Download FIG S5, TIF file, 1.1 MB. Copyright © 2022 Kundracik et al. 2022 Kundracik et al. https://creativecommons.org/licenses/by/4.0/ This content is distributed under the terms of the Creative Commons Attribution 4.0 International license.

The initial junctions correspond approximately to the two transmembrane domains in PopB (residues 170 to 201 and 232 to 258). Constructs B1 and B2, which replace the N-terminal and C-terminal extracellular portions of PopB with the corresponding regions of AopB, resulted in a weak tip-dependent phenotype and loss of function, respectively (Fig. 2B). We therefore decided to replace either the central portion of PopB (creating construct B3) or both the N- and C-terminal domains (construct B4). Construct B4 retained the tip-dependent phenotype of AopB. We therefore focused on further narrowing down the portions of the extracellular regions of AopB that clash with the wild-type PcrV needle tip. We subdivided the N- and C-terminal extracellular domains and replaced those regions with the homologous portions of AopB (constructs B5 and B6). While B5 was functional in the context of PcrV, construct B6 still required the hybrid Atip needle tip for function. Further reduction of the C-terminal substitution (constructs B7 and B8) and of the N-terminal substitution (Fig. S4) maintained the tip-dependent phenotype.

Our final chimeric construct, Bmix, had residues 114 to 150 and 312 to 330 of PopB replaced with the homologous segments of AopB (Fig. 2C). While neither substitution on its own displayed a significant defect in T3SS-mediated cytotoxicity, the combination of both substitutions resulted in Atip-dependent cell rounding, arguing that both segments are required for a contact with the tip domain. To verify this interaction, we assessed disulfide bond formation between cysteine mutant PopB(I117C) (PopB bearing a mutation of Ile to Cys at position 117) or PopB(S321C) paired with a tip domain cysteine mutant, PcrV(S202C) or a collar domain cysteine mutant, PcrV(Q87C) (Fig. 2D and E). Both PopB cysteine mutants were able to form a disulfide bond with PcrV(S202C) but not PcrV(Q87C), confirming that these regions of PopB interact with the needle tip protein tip domain. These data, together with the genetic rescue by the Atip needle tip, also argue that the loss of function of the Bmix protein in the context of wild-type PcrV is due to the loss of the interaction with the needle tip, rather than the result of misfolding of the chimeric Bmix protein.

In the course of mapping the region of incompatibility between AopB and PcrV, we noticed that many of the constructs that began with PopB and ended with AopB were inactive (Fig. S5). The converse fusions retained activity, suggesting that the choice of fusion joint was not to be blamed for the defect. Indeed, function was restored in sandwich fusions in which the PopB N- and C-termini were paired together (e.g., construct B4), suggesting that the mismatch interrupted a critical interaction between the N- and C-termini of PopB. Whether this interaction was intra- or intermolecular (i.e., needed for PopB dimer formation) was unclear. Additionally, we examined whether the AcrV hypervariable domain was responsible for the tip dependence of AopB. Deletion of the two 25-amino-acid helices unique to AcrV did not result in incompatibility with Bmix (Fig. S6), arguing that the incompatibility stemmed from a different, more conserved portion of the PcrV tip.

10.1128/mbio.02381-22.6 FIG S6 The nonhomologous segment of AcrV is not required for rescue of Bmix. Related to Fig. 2. (A) Residues 220 to 269 of AcrV, which have no homologous segment in PcrV, were deleted from the Atip construct to create the PcrV-AcrV chimera “Atip-trim.” Models of PcrV, Atip, and Atip-trim were created as described in the legend to Fig. 1. (B) The experiment was performed as described in the legend to Fig. 1, using background strains RP3624 (PcrV), RP6425 (Atip), and RP12517 (Atip-trim). Statistical significance was calculated by two-way ANOVA with the Tukey multiple-comparison test. Data bars are a summary of the results from three biological replicates with error bars showing standard deviations. ns, not significant (*P* > 0.05). Download FIG S6, TIF file, 0.9 MB. Copyright © 2022 Kundracik et al. 2022 Kundracik et al. https://creativecommons.org/licenses/by/4.0/ This content is distributed under the terms of the Creative Commons Attribution 4.0 International license.

### The PopB-tip domain interaction is not required for membrane insertion of PopB.

**FIG 3:**
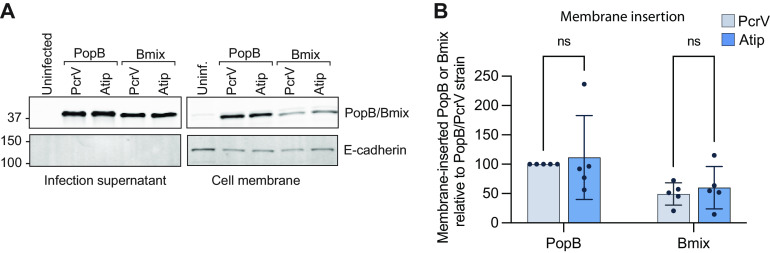
The Bmix-PcrV mismatch does not hinder membrane insertion of PopB. A549 cells were infected for 2 h with P. aeruginosa cells producing PopD and the indicated needle tip/B-translocator combination (background strains RP3670 and RP11222). The control strain lacks *pcrH*, *popB*, and *popD*. After infection, cells were washed with 1 M KCl to remove nonspecifically adhered protein, and membrane-inserted translocator proteins were extracted using Triton X-100 (see Materials and Methods). (A) Translocator proteins, as well as E-cadherin (fractionation/loading control), were detected by Western blotting in membrane extract and supernatant samples. The blot is representative of five independent experiments. The positions of molecular-weight size markers (in kilodaltons) are indicated. (B) Quantification of 5 biological replicates. The amount of PopB/Bmix is normalized to that of E-cadherin or EGFR. Error bars show standard deviations. Statistical significance was assessed with two-way ANOVA using the Sidak multiple-comparison test. ns, not statistically significant (i.e., *P* > 0.05).

We next sought to understand which stages of translocon assembly were affected by the PopB/PcrV mismatch. Since the needle tip was required for translocator insertion, we hypothesized that the tip domain interaction was critical for membrane insertion of PopB and PopD. We infected A549 epithelial cells with P. aeruginosa strains carrying the chimeric Bmix protein and either wild-type PcrV or chimeric Atip and assessed the amount of B-translocator in the host membrane by Western blotting. There was no significant difference in the amounts of membrane-inserted Bmix in the context of wild-type PcrV or chimeric Atip, indicating that the tip domain interaction was not required for membrane insertion of PopB (Fig. 3).

### Pore formation is hindered by disruption of the interaction between PopB and the tip domain of PcrV.

**FIG 4:**
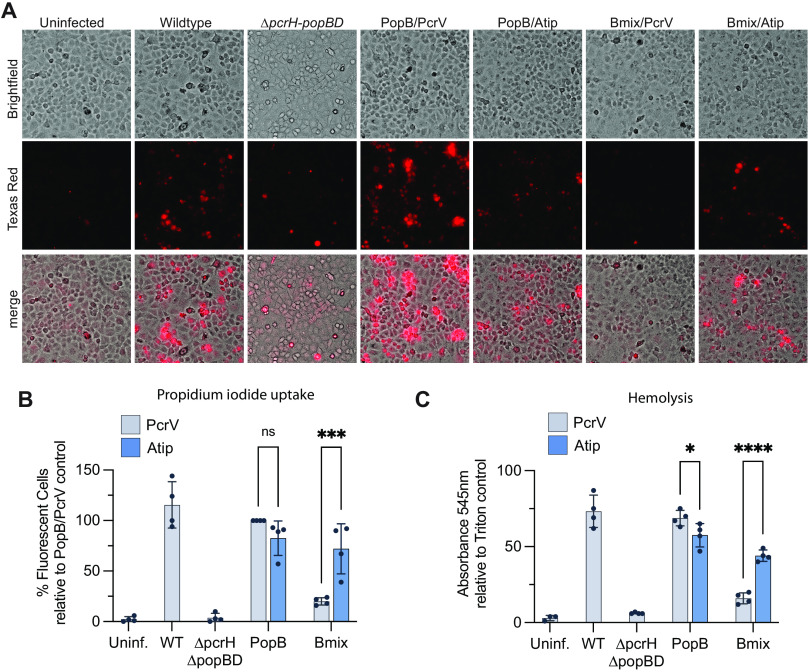
Pore formation is disrupted by mismatch between PopB and PcrV. (A, B) A549 cells were infected in the presence of propidium iodide with RP11946 (PAO1 *ΔexsE ΔexoSTY pcrV^+^ ΔpcrH-popBD*) or RP11948 [PAO1 *ΔexsE ΔexoSTY pcrV*::*acrV*(tip) *ΔpcrH-popBD*] producing PopD and the indicated translocators. RP2317 (PAO1 *ΔexsE ΔexoSTY pcrV^+^*) was the wild-type control. After 2 h, cells were washed and imaged (bright field and fluorescence with Texas red filter). (A) Representative micrographs from one biological replicate are shown. (B) Four biological replicates are quantified. Fluorescent cells were scored manually, and the results of each experiment were normalized to the results for the PopB/PcrV strain. Error bars show standard deviations. Statistical differences were analyzed with two-way ANOVA and the Sidak multiple-comparison test: *, *P* < 0.05;ns, not statistically significant (*P* > 0.05). (C) Sheep erythrocytes were pretreated with papain, which cleaves surface glycoproteins and exposes cryptic binding sites for P. aeruginosa hemagglutinin (53). The erythrocytes were infected for 1 h with strain RP3670 [PAO1 *ΔexsE exoS*(G/A−) *pcrV^+^ ΔpcrH-popBD*] or strain RP11222 [PAO1 *ΔexsE exoS*(G/A−) *pcrV*::*Atip ΔpcrH-popBD*] producing PopD and the indicated translocators. The erythrocyte suspension was then mixed, unbroken erythrocytes pelleted, and supernatant UV-Vis absorbance measured at 545 nm. The results of each experiment were normalized to the results for a Triton X-100-lysed control. *n* = 4 biological replicates. Statistical differences were analyzed with two-way ANOVA and the Sidak multiple-comparison test. Significance thresholds: *, *P* < 0.05; ****, *P* < 0.0001; ns, not statistically significant (*P* > 0.05).

To investigate whether disruption of the PopB-PcrV interaction would affect pore formation, A549 cells were infected in the presence of propidium iodide (PI), which is small enough (668 Daltons) to enter cells through translocation pores (34–36). There was no significant difference in PI uptake between strains producing wild-type PopB and either PcrV or Atip, reiterating our finding from the cytotoxicity assay that PopB can be paired with either version of the needle tip protein (Fig. 4A and B). On the other hand, the Bmix/PcrV strain averaged less than 25% the amount of PI uptake as the PopB/PcrV strain, but this defect was significantly corrected in the Bmix/Atip strain. We employed a pore formation assay in sheep red blood cells to corroborate these findings (Fig. 4C). In the hemolysis assay, Bmix/Atip performed significantly better than Bmix/PcrV. We also observed a small but statistically significant defect for PopB/Atip compared to PopB/PcrV, consistent with the importance of this interaction for pore formation. Together, these findings indicate that a mismatch between PopB and the tip domain of PcrV impedes the formation of the translocation pore.

### Defective translocation in the mismatched Bmix-PcrV strain was not overcome by bypassing secretion regulation.

**FIG 5:**
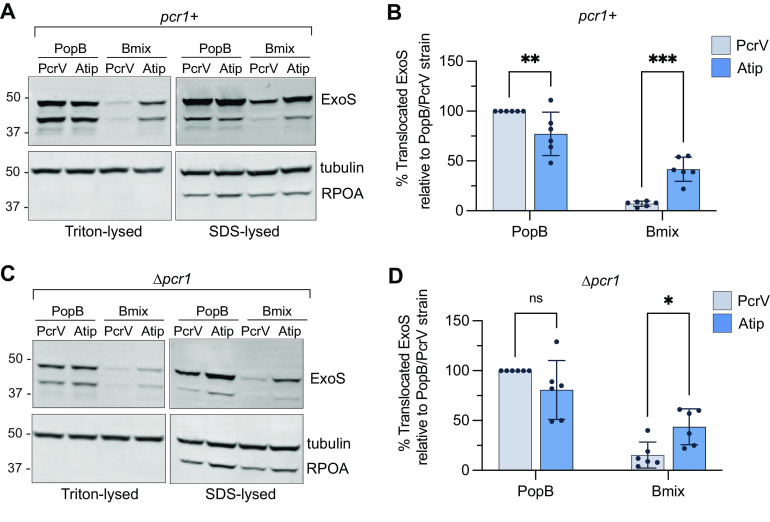
Deletion of the secretion regulator Pcr1 does not restore translocation for the translocator-mismatched (Bmix-PcrV) strain. A549 cells were infected for 2 h with strain RP3670 [PAO1 *ΔexsE exoS*(G/A−) *pcrV^+^ ΔpcrH-popBD*], strain RP11222 [PAO1 *ΔexsE exoS*(G/A−) *pcrV*::*Atip ΔpcrH-popBD*], strain RP6370 [PAO1 *ΔexsE exoS*(G/A−) *pcrV^+^ ΔpcrH-popBD Δpcr1*], or strain RP11226 [PAO1 *ΔexsE exoS*(G/A−) *pcrV*::*Atip ΔpcrH-popBD Δpcr1*] complemented with *popD* and the indicated B-translocator, along with both translocator chaperones, *pcrH* and *acrH*. The amount of translocated ExoS was assessed by lysing the host cells with either Triton X-100, which lyses the eukaryotic cell membrane but does not lyse the bacteria, or lysing with SDS, which lyses both the eukaryotic cell and peripherally attached bacteria. ExoS was also monitored in supernatant samples and in bacteria collected from the supernatant after infection (shown in Fig. S7). The presence of ExoS, tubulin (host cell cytoplasmic content), and RNA polymerase subunit α (RPOA, bacterial cytoplasmic content) was assessed by Western blotting. (A and C) Western blots of *pcr1^+^* strains (A) and *Δpcr1* strains (C). The amount of ExoS translocated into A549 cells is shown by the Triton-lysed samples. The lower-molecular-weight band is attributable to intracellular cleavage of ExoS (59). The positions of molecular-weight size markers (in kilodaltons) are indicated. SDS-lysed samples show the total amount of ExoS both translocated into host cells and remaining in attached bacteria. (B and D) Quantification of ExoS translocation in Triton-lysed samples for *pcr1^+^* strains (B) and *Δpcr1* strains (D). The amount of translocated ExoS is normalized to the amount of tubulin in the same strain and to the amount of ExoS in the strain producing PopB/PcrV. *n* = 6 biological replicates. Error bars show standard deviations. Statistical differences were analyzed with two-way ANOVA and the Sidak multiple-comparison test. Significance thresholds: *, *P* < 0.05; **, *P* < 0.005; ***, *P* < 0.0005; ns, not statistically significant (*P* > 0.05).

A hallmark of all T3SS is that secretion of effector proteins is triggered by host cell contact (37, 38). The nature of the trigger is unknown but, in P. aeruginosa, involves a conformational change in the translocon that alters a contact between PopD and the PcrV collar domain (25). We set out to test whether the mismatch between PcrV and Bmix interrupts this triggering mechanism. In P. aeruginosa, the PopN-Pcr1 complex prevents effector secretion before host cell contact (39). Deletion of *pcr1* results in constitutive effector secretion, regardless of host cell contact (25). To test whether the PopB-PcrV interaction was important for secretion regulation, we measured the translocation of the effector ExoS in strains lacking the secretion regulator Pcr1. If the PopB-PcrV interaction was primarily required for sensing host cell contact, deletion of *pcr1* should restore translocation in the Bmix/PcrV strain, since a *pcr1* mutant no longer required host cell contact to initiate effector export. Consistent with the cytotoxicity assays, translocation of ExoS was impaired in the Bmix/PcrV strain but significantly restored in the Bmix/Atip strain (Fig. 5). However, deletion of *pcr1* was not sufficient to restore ExoS translocation in the mismatched Bmix/PcrV strain. Secretion into the extracellular milieu was not affected (Fig. S7). Taken together, these results indicated that the translocation defect of the mismatched Bmix/PcrV strain was primarily due to the defect in pore formation. An additional defect in host cell sensing cannot be ruled out since inhibition of pore assembly could be masking this step.

10.1128/mbio.02381-22.7 FIG S7 Supernatant samples for the translocation assay. Related to Fig. 5. The experiment was performed as described in the legend to Fig. 5 using background strains RP3670 (PcrV), RP11222 (Atip), RP6370 (PcrV *Δpcr1*), and RP11226 (Atip *Δpcr1*). (A) Schematic diagram of experimental design. (B) Supernatant samples show the amounts of ExoS secreted before host cell contact. Deletion of the secretion regulator *pcr1* causes premature secretion of ExoS. (C) Bacterial samples show the amounts of ExoS contained in the bacteria cytosol at the end of the infection. Download FIG S7, TIF file, 1.9 MB. Copyright © 2022 Kundracik et al. 2022 Kundracik et al. https://creativecommons.org/licenses/by/4.0/ This content is distributed under the terms of the Creative Commons Attribution 4.0 International license.

## DISCUSSION

Mapping interactions among translocator proteins has been challenging. Biochemical assays have been used to demonstrate interactions by using purified pore-forming translocator proteins (18, 40). Recently, a combination of accessibility of cysteine mutants to chemical modification and disulfide bond formation between adjacent residues was used to map interactions in the Shigella flexneri translocation pore assembled in the plasma membrane of infected cells (28, 41, 42). Here, we used a genetic approach to identify several functionally important interactions that were disrupted by pairing P. aeruginosa and A. hydrophila translocator proteins (Fig. 1). First, our data indicated that AopD failed to interact productively with the P. aeruginosa needle tip protein PcrV. AopD, just like YopD, harbors a phenylalanine residue at a position corresponding to alanine 292 of PopD, which we had shown previously disrupts a critical contact with the collar domain of PcrV (25). However, unlike the YopD-PcrV incompatibility, producing AopD in the context of a hybrid needle tip in which the collar domain of PcrV was replaced with the corresponding region of AcrV (Acollar) only partially restored function. Since AopD fully supported translocation in the context of AcrV, this result argued that AopD made two contacts with the needle tip: one with the collar domain, and a second interaction with the tip domain of the needle tip protein. Second, while mapping the incompatibility between AopB and PcrV, we discovered an internal N- and C-termini of PopB that was also disrupted in PopB-AopB hybrids (Fig. S5). Function could be restored by having both the N-terminus and C-terminus of the hybrid protein derive from PopB. The interaction that was disrupted in the PopB-AopB hybrids could be intramolecular, i.e., required for the folding of individual PopB monomers, or intermolecular, i.e., required for PopB oligomerization. Further research will be needed to distinguish between these possibilities. Finally, we found that the tip domain of the needle tip protein interacted with two regions of the B-translocator (Fig. 2). The needle tip-dependent translocation defect was only evident when both regions of PopB (residues 114 to 150 and 312 to 330) were replaced with the corresponding regions of AopB (resulting in the construct Bmix), suggesting that the two regions both interacted with the needle tip and that a partial substitution was not divergent enough to block the contact with the P. aeruginosa needle tip protein PcrV. As with the internal PopB interaction, the two interacting regions we identified here could be involved in an intermolecular interaction that allows a PopB monomer to interact with the needle tip. However, PopB can dimerize (25), and so the two regions could also be part of an epitope that is formed by two adjacent PopB monomers, which is then recognized by the needle tip. A better structural understanding of the translocon will be necessary to resolve this question.

Antibodies directed against the tip of PcrV are protective *in vivo* and have been used in clinical trials to treat Pseudomonas infections (43–45). Since this portion of the needle tip had not been implicated in a particular translocon function, we decided to delve more deeply into the interaction between PopB and the tip domain of PcrV. Using a construct that specifically disrupts this interaction (Bmix), as well as a hybrid needle tip that restores translocation in the context of Bmix (Atip), we assessed several aspects of translocon function to pinpoint the role of this interaction in effector translocation. First, we assessed the ability of PcrV to insert PopB or Bmix into the host cell membrane. While we recovered less Bmix than PopB in these experiments, the amount was not affected by the needle tip, demonstrating that the Bmix-PcrV incompatibility did not affect Bmix insertion (Fig. 3). We next assessed the ability of P. aeruginosa to form pores in membranes by detecting translocon-dependent uptake of propidium iodide. Unlike in the translocon insertion experiments, we observed a defect in pore formation by the Bmix/PcrV strain, which was rescued in the Bmix/Atip strain (Fig. 4). Taken together, these data argue that the interaction identified here is required for the assembly of the translocation pore. We also assayed downstream steps in translocation by detecting translocated ExoS protein in lysates of infected epithelial cells. While we could recapitulate the tip-dependent translocation phenotype of bacteria producing the Bmix translocator protein, this defect could not be restored by removing a negative regulator of effector secretion, Pcr1, from the system (Fig. 5). Restoration of effector injection by removing Pcr1 would have indicated that the Bmix-PcrV mismatch interfered with host cell sensing. The defect in pore assembly appears to be the primary block in translocon function incurred by the Bmix-PcrV mismatch. However, an additional role of this interaction in the docking of the tip to the translocation pore or in host cell sensing cannot be ruled out, since a block in an earlier step of the translocation process could mask a defect in these later steps.

**FIG 6:**
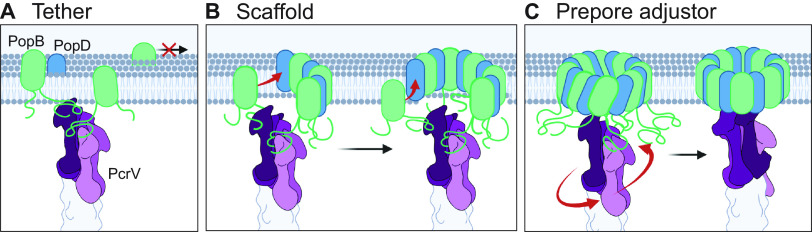
Models of PcrV-assisted translocation pore formation. (A) Tether model: PcrV prevents lateral diffusion of PopB and PopD in the membrane, encouraging oligomerization. (B) Scaffold model: the pore proteins could oligomerize around the PcrV needle tip as they are secreted. (C) Prepore model: PopB and PopD could form a prepore structure which is converted by PcrV to its final form. Schematic figures were created using BioRender.com.

Our genetic analysis of translocon function has uncovered a novel step in the translocon assembly process: needle tip-guided formation of the translocation pore. We envision three different models for this activity (Fig. 6). First, the needle tip could simply serve as a tether for inserted translocator proteins, increasing their local concentration and thereby driving pore formation. Second, the needle tip could serve as a scaffold to actively fold and assemble inserted translocator proteins into the nascent pore. Third, the translocator proteins could insert and assemble into a prepore, which is opened through the interaction with the needle tip. The latter is reminiscent of an assembly pathway that was suggested for the Shigella T3SS, where it was proposed that IpaB may insert into the plasma membrane as a prepore, based on the observation that an *ipaC* null mutant still retains residual ability to lyse red blood cells (11, 46). Which model is correct, or whether multiple aspects of these models (e.g., tethering and chaperoned folding/assembly) play a role in the assembly of the translocation pore awaits further research. It is also not clear whether PopD similarly relies on the needle tip for assembly into the translocation pore. Intriguingly, our genetic analysis indicates that AopD also requires the tip domain of AcrV for its function. While we have not fully mapped this interaction yet, it will be interesting to see if this interaction is also specifically required for pore formation. Notably, purified PopB and PopD form oligomeric complexes on their own *in vitro*, as well as heterooligomeric complexes if administered simultaneously. However, PopD complexes cannot be disrupted through the subsequent addition of PopB (18), arguing that this homomeric complex would likely have to be avoided during assembly on the cell surface. Perhaps needle tip-guided pore assembly avoids this dead end? Our results also have implications for interpreting *in vitro* models of translocation pore formation. In such studies, PopB and PopD are used to form pores in lipid vesicles, but in the absence of the needle tip. If the contribution of the needle tip is to increase the local concentration of translocator proteins, then this function could likely be overcome by increasing the concentration of translocator proteins in those *in vitro* reactions. However, if the needle tip serves as a scaffold that helps organize the pore, then this raises the question of how faithfully translocation pores assembled *in vitro* recapitulate pores formed by infecting bacteria in host cells. Clearly, assembling this key interface between the host cell and the bacterium is more complex than initially anticipated.

## MATERIALS AND METHODS

### Bacterial strains, cells, and growth conditions.

All bacterial strains used in the study are listed in Table S1. E. coli strains were grown in LB medium (10 g/L tryptone, 5 g/L yeast extract, 5 g/L NaCl). P. aeruginosa strains were grown in high-salt LB (LB with 11.7 g/L NaCl, 5 mM MgCl_2_, and 0.5 mM CaCl_2_). Plasmids were retained with 15 μg/mL gentamicin. The expression of plasmid-borne genes under the control of the *lacUV5* promoter was induced with 100 μM IPTG (isopropyl β-d-1-thiogalactopyranoside).

A549 cells (ATCC CCL-185) were grown in RPMI 1640 supplemented with 10% fetal bovine serum (FBS) (RP10 medium) at 37°C with 5% CO_2_. The cells were maintained with penicillin/streptomycin. Before infection, A549 cells were rinsed once with Dulbecco’s phosphate-buffered saline (DPBS), and the medium was exchanged with RP10 without antibiotics [RP10(−)].

### Plasmid and strain construction.

The plasmids used in this study are listed in Table S1. The parent strain was PAO1 (47). Purified DNA from Aeromonas hydrophila strain AH-3 (48) was used as the template for *acrH*, *aopB*, *aopD*, and *acrV*. Plasmids were constructed using standard molecular biology techniques. Wild-type and chimeric translocator genes were cloned by splicing by overlap extension (SOE)-PCR using the primers listed in Table S2. PCR products were digested and ligated into plasmid pEXG2 (allelic exchange vector), pPSV37 (a plasmid that can replicate in P. aeruginosa with the *lacUV5* promoter and *lacIq*), or pPGEH (a plasmid that can replicate in P. aeruginosa with the T3SS *pcrG* promoter). Mutations in the P. aeruginosa chromosome were introduced by allelic exchange as described previously (49). pPG-*popD* was generated by amplifying three fragments from pP37-*popD* (50) using primers dblatoOri/pGtoRepA, dblatoGent/dlacItoGent, and dlacItoTerm/pGtoPopD that were then combined by Gibson assembly (51), thereby removing the *lacI* gene and remnants of the *bla* open reading frame (ORF) in pPSV37, as well as replacing the lacUV5 promoter with the promoter upstream from *pcrG* and introducing a BbvCI site between the gentamicin resistance gene and the replication origin. pPGEH was generated by amplifying the vector backbone of pPG-*popD* using primers pGpolyFor and pGpolyRev and recircularizing the resultant PCR product using Gibson assembly.

10.1128/mbio.02381-22.9 TABLE S2 Primers used for cloning in this study. Download Table S2, XLSX file, 0.02 MB. Copyright © 2022 Kundracik et al. 2022 Kundracik et al. https://creativecommons.org/licenses/by/4.0/ This content is distributed under the terms of the Creative Commons Attribution 4.0 International license.

### Cytotoxicity.

Cytotoxicity assays were performed as described previously (25). Briefly, A549 cells were seeded in a 24-well plate at a density of 7.5e4 cells/well. The next day, A549 cells were rinsed twice with DPBS and the medium was replaced with RP10(−) supplemented with 100 μM IPTG. A549 cells were infected with mid-log-phase bacteria at a multiplicity of infection (MOI) of 25. The infection was stopped by fixation with formaldehyde after 2 h, except for the kinetics experiments, which were stopped at various time points up to 3 h. Round versus flat (“healthy”) cells were counted manually by low-power phase-contrast microscopy.

### Disulfide cross-linking.

Disulfide cross-linking assays were as described previously (25), with some modifications. Pseudomonas strains producing cysteine mutants of PopB and/or PcrV were used to infect A549 epithelial cells for 2 h in the presence of 25 μM copper-(1,10)-phenanthroline. After infection, the cells were washed with 10 mM iodoacetamide for 10 min to alkylate free cysteine residues. The cells were rinsed with PBS-MC (phosphate buffered saline supplemented with 5 mM MgCl_2_ and 0.5 mM CaCl_2_) and then harvested into 1 mL PBS-MC with 2 mM phenylmethylsulfonyl fluoride (PMSF) and Roche cOmplete mini protease inhibitor. The cells were pelleted (5 min at 2,300 × *g*). The cell membranes were solubilized with 0.1% Triton X-100 in the presence of 2 mM PMSF for 15 min at room temperature. The cellular debris was pelleted (3 min at 9,300 × *g*), and the supernatant was mixed with 4× SDS sample buffer with or without dithiothreitol (DTT) as indicated. The samples were heated for 10 min at 80°C and analyzed by Western blotting.

### A549 membrane insertion.

A549 cells were infected with strain PAO1 harboring the indicated combinations of translocator proteins. After infection, supernatant samples were cleared of bacteria by centrifugation. The A549 cells were rinsed in high-salt buffer (PBS-MC with 1 M KCl) to remove peripherally associated proteins from the membrane. To release intracellular proteins, the A549 cells were treated for 30 min with streptolysin O (Millipore) preactivated with 10 mM DTT. The cells were harvested by scraping and treated with Triton X-100. Cellular debris was removed by centrifugation, and the solubilized membrane was analyzed by Western blotting. Blots were probed for PopB, and the membrane protein E-cadherin or epidermal growth factor receptor (EGFR) was used as a loading control.

### Propidium iodide uptake.

The day prior to infection, a 24-well plate was seeded with 9e5 A549 cells/well. The day of infection, the medium was changed to RPMI 1640 (without phenol red) supplemented with 6 μg/mL propidium iodide (Biotium). Cells were infected at an MOI of 100 for 2 h and then rinsed twice with PBS-MC. The unfixed cells were imaged with a Cytation 5 imager (BioTek) using a 20× objective for bright-field and Texas red fluorescence imaging. Red cells were manually counted.

### Hemolysis.

Following the previously described protocol (52), sheep erythrocytes in sodium citrate buffer (Quad Five) were washed several times with PBS until the supernatant was clear and then treated with 0.1% papain and 0.01% cysteine for 30 min to promote adhesion by P. aeruginosa (53). Erythrocytes were washed again and resuspended in RPMI 1640 (without phenol red) to a concentration of 5e8 cells/mL. P. aeruginosa strains at mid-log phase were pelleted and resuspended in PBS-MC, the optical density at 600 nm (OD_600_) was measured, and bacteria were resuspended to a concentration of 2.5e9 cells/mL. Bacteria and red blood cells were mixed thoroughly 1:1 (MOI of 5). Bacteria and erythrocytes were centrifuged (2,000 × *g* for 5 min). After 2 h at 37°C, the infection mixtures were resuspended and unbroken cells were removed by centrifugation. The absorbance at 545 nm was read with a BioTek Synergy HT plate reader. Hemolysis was calculated as follows: % hemolysis = (*A*_sample_ − *A*_PBS blank_)/(*A*_Triton_ − *A*_PBS blank_).

### Translocation.

ExoS regulates its own secretion through a feedback loop (30, 34). To remove feedback regulation from the equation, we used strains producing a version of ExoS in which the enzymatic activities have been inactivated by point mutations (30). The day before the experiment, A549 cells were seeded in 10-cm^2^ tissue culture-treated dishes at 1.5e6 cells/dish. A549 epithelial cells were infected with mid-log-phase P. aeruginosa cells for 2 h at an MOI of 25, washed three times with PBS-MC, and rinsed with 1 mL of 250 μg/mL proteinase K in PBS-MC. The protease solution was removed promptly, and the cells were incubated at room temperature for 15 min to digest extracellular protein. Protease-treated cells were resuspended in 1 mL of PBS-MC with 2 mM PMSF, pelleted (3 min at 8,000 × *g*), and resuspended in 95 μL of PBS-MC with 0.1% Triton X-100. After incubation on ice for 15 min, 45 μL of the cell suspension was removed and mixed with 15 μL of 4× SDS sample buffer (SDS sample). The remaining cells were pelleted, and 45 μL of supernatant was removed and combined with 15 μL of 4× SDS sample buffer (Triton sample). Samples were heated for 10 min at 95°C, separated by SDS-PAGE, and analyzed by Western blotting. Membranes were probed with antibodies directed against ExoS, tubulin, and RNA polymerase α (RpoA).

### Western blotting.

Samples were separated on 10% SDS-PAGE gels (Bio-Rad) alongside a molecular-weight marker (Bio-Rad precision plus protein dual color standard). Samples were transferred to Immobilon-FL PVDF (Millipore) for fluorescence imaging or Immobilon-PSQ PVDF (Millipore) for chemiluminescence imaging. For fluorescence detection, blots were incubated with LI-COR anti-mouse secondary antibody for detection at 700 nm or anti-rabbit secondary antibody for detection at 800 nm, both at 1:10,000 concentration, and imaged with a LI-COR Odyssey system. Densitometry analysis was performed with LI-COR Odyssey software and ImageJ. For chemiluminescence detection, blots were incubated with horseradish peroxidase (HRP)-conjugated goat anti-rabbit or goat anti-mouse secondary antibodies. Detection was performed with chemiluminescent substrate (Advansta Sirius) with image detection on a GE ImageQuant LAS 4000 imager.

### Statistical analysis.

Statistical analysis was performed in GraphPad Prism using at least three biological replicates performed on independent days using independent bacterial cultures. Statistical tests are listed in figure legends.
