# Supplementary material for: PopB-PcrV Interactions Are Essential for Pore Formation in the Pseudomonas aeruginosa Type III Secretion System Translocon
Source: mBio. 2022 Sep 26;13(5):e02381-22. doi: 10.1128/mbio.02381-22 (PMC9600203; doi:10.1128/mbio.02381-22)
Supplement: TABLE S1 [file mbio.02381-22-s0008.pdf]

Table S1. Strains and Plasmids

Strain #	Genotype	Ref.
RP2318	PAO1F Δ*exsE* Δ*exoT* Δ*exoY*	Cisz 2008
RP3624	PAO1F Δ*exsE* Δ*exoT* Δ*exoY* Δ*pcrHpopBD pcrV*+	Armentrout 2016
RP6425	PAO1F Δ*exsE* Δ*exoT* Δ*exoY* Δ*pcrHpopBD* Δ*pcrV*::*pcrV*(*acrV*tip)	This study
RP6166	PAO1F Δ*exsE* Δ*exoT* Δ*exoY* Δ*pcrHpopBD* Δ*pcrV*::*acrV*(N)-*pcrV*(C)	This study
RP7595	PAO1F Δ*exsE* Δ*exoT* Δ*exoY* Δ*pcrHpopBD* Δ*pcrGV*::*acrGV*	This study
RP3223	PAO1F Δ*exsE* Δ*exoT* Δ*exoY* Δ*pcrV*2	This study
RP2349	PAO1F Δ*exsE* Δ*exoT* Δ*exoY* *exoS*(R146K/E379D/E381D)	Cisz 2008
RP3670	PAO1F Δ*exsE* Δ*exoT* Δ*exoY* *exoS*(R146K/E379D/E381D) Δ*pcrHpopBD pcrV*+	Armentrout 2016
RP11222	PAO1F Δ*exsE* Δ*exoT* Δ*exoY* *exoS*(R146K/E379D/E381D) Δ*pcrHpopBD* Δ*pcrV*::*pcrV*(*acrV*tip)	This study
RP6370	PAO1F Δ*exsE* Δ*exoT* Δ*exoY* *exoS*(GAP-/ADPR-) Δ*pcrHpopBD pcrV*+	Armentrout 2016
RP11226	PAO1F Δ*exsE* Δ*exoT* Δ*exoY* *exoS*(GAP-/ADPR-) Δ*pcrHpopBD* Δ*pcrV*::*pcrV*(*acrV*tip)	This study
RP2317	PAO1F Δ*exsE* Δ*exoS* Δ*exoT* Δ*exoY*	Cisz 2008
RP11946	PAO1F Δ*exsE* Δ*exoS* Δ*exoT* Δ*exoY* Δ*pcrHpopBD pcrV*+	This study
RP11948	PAO1F Δ*exsE* Δ*exoS* Δ*exoT* Δ*exoY* Δ*pcrHpopBD* Δ*pcrV*::*pcrV*(*acrV*tip)	This study
RP12517	PAO1F Δ*exsE* Δ*exoT* Δ*exoY* S+ Δ*pcrHpopBD* Δ*pcrV*::*pcrV*(*acrV*tip-trim)	This study
RP12661	PAO1F Δ*exsE* Δ*exoT* Δ*exoY* *exoS*(GAP-/ADPR-) Δ*pcrHpopBD pcrV*(Q87C)	This study
RP12598	PAO1F Δ*exsE* Δ*exoT* Δ*exoY* *exoS*(GAP-/ADPR-)Δ*pcrHpopBD pcrV*(S202C)	This study

Plasmid name	Description	Hybrid	Reference
pPSV37	*colE1* origin, *gentR*, PA origin, *oriT*, *lacUV5* promoter, *lacIq*		Lee 2010
pEXG2	allelic exchange vector, *colE1* origin, *oriT*, *gentR*, *sacB*		Rietsch 2005
pPGEH	derived from pPSV37, *pcrG* promoter, *gentR*, BbvCI site, polylinker, *colE1* origin, PA origin		This study
pEXG2-Δ*pcrH*Δ*popB*Δ*popD*	allelic exchange plasmid deleting *pcrH* codon 5 to *popD* codon 268		Tomalka 2012
pEXG2-*pcrV*(*acrV*tip)	allelic exchange plasmid for *pcrV*::Atip		This study
pEXG2-*acrV*(N)-*pcrV*(C)	allelic exchange plasmid for *pcrV*::Acollar		This study
pEXG2-Δ*pcrV*::*acrV*	allelic exchange plasmid for *pcrV*::AcrV		This study
pEXG2-Δpcr1	allelic exchange plasmid deleting codons 10–81 of pcr1		Tomalka 2012
pEXG2-Atip-trim	allelic exchange plasmid for *pcrV*::Atip-trim		This study
pEXG2-Δ*pcrV*2	allelic exchange plasmid deleting *pcrV* codons 4-180		Cisz 2008
pP37-*acrHpcrH-popBD*	Plasmid encoding *acrH*, *pcrH*, *popB* and *popD* under the control of the lacUV5 promoter in pPSV37		Armentrout 2016
pP37-*acrHpcrH-aopB*D	Plasmid encoding *acrH*, *pcrH*, *aopB* and *aopD* under control of the lacUV5 promoter in pPSV37		This study
pP37-*acrHpcrH-popB-aopD*	Plasmid encoding *acrH*, *pcrH*, *popB* and *aopD* under control of the lacUV5 promoter in pPSV37		This study
pP37-*acrHpcrH-aopBpopD*	Plasmid encoding *acrH*, *pcrH*, *aopB* and *popD* under control of the lacUV5 promoter in pPSV37		This study
pP37-*acrHpcrHpopD*	Plasmid encoding *acrH*, *pcrH*, and *popD* under control of the lacUV5 promoter in pPSV37		This study
pP37-*acrHpcrHpopB*	Plasmid encoding *acrH*, *pcrH* and *popB* under control of the lacUV5 promoter in pPSV37		This study

pP37-*acrHpcrH*-APB 169-*popD*	Plasmid encoding *acrH*, *pcrH*, a *aopB*(1-161G)-*popB*(169K-390) hybrid and *popD* under the control of the lacUV5 promoter in pPSV37, hybrid "B1"	B1	This study
pP37-*acrHpcrH*-PAB 250-*popD*	Plasmid encoding *acrH*, *pcrH*, *popD* and a *popB*(1-250G)-*aopB*(244S-390) hybrid under the control of the lacUV5 promoter in pPSV37, hybrid "B2"	B2	This study
pP37-*acrHpcrH*-PAPB 168-251-*popD*	Plasmid encoding *acrH*, *pcrH*, *popD* and a *popB*(1-168A)-*aopB*(162K-243G)-*popB*(251G-390) hybrid under the control of the lacUV5 promoter in pPSV37, hybrid "B3"	B3	This study
pP37-*acrHpcrH*-APAB 183-250-*popD*	Plasmid encoding *acrH*, *pcrH*, *popD* and a *aopB*(1-175I)-*popB*(183G-250G)-*aopB*(244S-390) hybrid under the control of the lacUV5 promoter in pPSV37, hybrid "B4"	B4	This study
pP37-*acrHpcrH*-APAPB 115-250-298-*popD*	Plasmid encoding *acrH*, *pcrH*, *popD* and a *aopB*(1-107L)-*popB*(115A-250G)-*aopB*(244S-297V)-*popB*(298-390) hybrid under the control of the lacUV5 promoter in pPSV37, hybrid "B5"	B5	This study
pP37-*acrHpcrH*-PAPAB 114-169-297-*popD*	Plasmid encoding *acrH*, *pcrH*, *popD* and a *popB*(1-114L)-*aopB*(108G-161G)-*popB*(169K-296H)-*aopB*(270A-390) hybrid under the control of the lacUV5 promoter in pPSV37, hybrid "B6"	B6	This study
pP37-*acrHpcrH*-PAPAPB 114-169-297-333-*popD*	Plasmid encoding *acrH*, *pcrH*, *popD* and a *popB*(1-114L)-*aopB*(108G-161G)-*popB*(169K-296H)-*aopB*(270A-332A)-*popB*(333D-390) hybrid under the control of the lacUV5 promoter in pPSV37, hybrid "B7"	B7	This study
pP37-*acrHpcrH*-PAPAPB 114-169-312-330-*popD*	Plasmid encoding *acrH*, *pcrH*, *popD* and a *popB*(1-114L)-*aopB*(108G-161G)-*popB*(169K-311V)-*aopB*(312S-332A)-*popB*(333D-390) hybrid under the control of the lacUV5 promoter in pPSV37,hybrid "B8"	B8	This study
pP37-*acrHpcrH*-PAPB 114-150-*popD*	Plasmid encoding *acrH*, *pcrH*, *popD* and a *popB*(1-114L)-*aopB*(108G-143K)-*popB*(150K-390) hybrid under the control of the lacUV5 promoter in pPSV37, hybrid "B9"	B9	This study
pP37-*acrHpcrH*-PAPB 312-330-*popD*	Plasmid encoding *acrH*, *pcrH*, *popD* and a *popB*(1-311V)-*aopB*(312S-330A)-*popB*(331Q-390) hybrid under the control of the lacUV5 promoter in pPSV37, hybrid "B10"	B10	This study
pP37-*acrHpcrH*-PAPAPB 114-150-312-330-*popD*	Plasmid encoding *acrH*, *pcrH*, *popD* and a *popB*(1-114L)-*aopB*(108G-143K)-*popB*(150K-311V)-*aopB*(312S-330A)-*popB*(331Q-390) hybrid under the control of the lacUV5 promoter in pPSV37, hybrid "Bmix"	Bmix	This study
pP37-*acrHpcrH*-PAB 40-*popD*	Plasmid encoding *acrH*, *pcrH*, *popD* and a *popB*(1-40L)-*aopB*(38T-390) hybrid under the control of the lacUV5 promoter in pPSV37, hybrid "B11"	B11	This study
pP37-*acrHpcrH*-PAPAPB 114-183-312-330-*popD*	Plasmid encoding *acrH*, *pcrH*, *popD* and a *popB*(1-114L)-*aopB*(108G-175I)-*popB*(183G-311V)-*aopB*(312S-330A)-*popB*(331Q-390) hybrid under the control of the lacUV5 promoter in pPSV37, hybrid "B12"	B12	This study
pP37-*acrHpcrH*-PAPAPB 131-183-312-330-*popD*	Plasmid encoding *acrH*, *pcrH*, *popD* and a *popB*(1-131T)-*aopB*(126I-175I)-*popB*(183G-311V)-*aopB*(312S-330A)-*popB*(331Q-390) hybrid under the control of the lacUV5 promoter in pPSV37, hybrid "B13"	B13	This study
pP37-*acrHpcrH*-PAPAPB 131-169-312-330-*popD*	Plasmid encoding *acrH*, *pcrH*, *popD* and a *popB*(1-131T)-*aopB*(125D-175I)-*popB*(183G-311V)-*aopB*(312S-330A)-*popB*(331Q-390) hybrid under the control of the lacUV5 promoter in pPSV37, hybrid "B14"	B14	This study
pP37-*acrHpcrH*-PAPAPB 151-183-312-330-*popD*	Plasmid encoding *acrH*, *pcrH*, *popD* and a *popB*(1-151I)-*aopB*(145K-175I)-*popB*(183G-311V)-*aopB*(312S-330A)-*popB*(331Q-390) hybrid under the control of the lacUV5 promoter in pPSV37, hybrid "B15"	B15	This study
pP37-*acrHpcrH*-PAB 114-*popD*	Plasmid encoding *acrH*, *pcrH*, *popD* and a *popB*(1-114L)-*aopB*(108G-390 hybrid under the control of the lacUV5 promoter in pPSV37, hybrid "B16"	B16	This study
pP37-*acrHpcrH*-PAB 168-*popD*	Plasmid encoding *acrH*, *pcrH*, *popD* and a *popB*(1-168A)-*aopB*(162K-390) hybrid under the control of the lacUV5 promoter in pPSV37, hybrid "B17"	B17	This study
pP37-*acrHpcrH*-PAB 297-*popD*	Plasmid encoding *acrH*, *pcrH*, *popD* and a *popB*(1-296H)-*aopB*(270A-390) hybrid under the control of the lacUV5 promoter in pPSV37, hybrid "B18"	B18	This study

pP37-*acrHpcrH*-PAB 330-*popD*	Plasmid encoding *acrH*, *pcrH*, *popD* and a *popB*(1-330R)-*aopB*(331Q-390) hybrid under the control of the lacUV5 promoter in pPSV37, hybrid "B19"	B19	This study
pP37-*acrHpcrH*-APB 115-*popD*	Plasmid encoding *acrH*, *pcrH*, *popD* and a *aopB*(1-107L)-*popB*(115A-390) hybrid under the control of the lacUV5 promoter in pPSV37, hybrid "B20"	B20	This study
pP37-*acrHpcrH*-APB 251-*popD*	Plasmid encoding *acrH*, *pcrH*, *popD* and a *aopB*(1-243G)-*popB*(251G-390) hybrid under the control of the lacUV5 promoter in pPSV37, hybrid "B21"	B21	This study
pP37-*acrHpcrH*-APB 298-*popD*	Plasmid encoding *acrH*, *pcrH*, *popD* and a *aopB*(1-297V)-*popB*(298L-390) hybrid under the control of the lacUV5 promoter in pPSV37, hybrid "B22"	B22	This study
pP37-*acrHpcrH*-APB 323-*popD*	Plasmid encoding *acrH*, *pcrH*, *popD* and a *aopB*(1-322V)-*popB*(323F-390) hybrid under the control of the lacUV5 promoter in pPSV37, hybrid "B23"	B23	This study
pP37-*acrHpcrH*-APB 369-*popD*	Plasmid encoding *acrH*, *pcrH*, *popD* and a *aopB*(1-368M)-*popB*(369I-390) hybrid under the control of the lacUV5 promoter in pPSV37, hybrid "B24"	B24	This study
pPGEH-*acrHpcrH-popBD*	Plasmid encoding *acrH*, *pcrH*, *popB* and *popD* under control of the *pcrG* promoter		This study
pPGEH-*acrHpcrH*-Bmix-*popD*	Plasmid encoding *acrH*, *pcrH*, *popD* and a *popB*(1-114L)-*aopB*(108G-143K)-*popB*(150K-311V)-*aopB*(312S-330A)-*popB*(331Q-390) hybrid under control of the *pcrG* promoter		This study
pP37-*acrHpcrH*-*popB*(Q135C)-*popD*	Plasmid encoding *acrH*, *pcrH*, *popB*(Q135C) and *popD* under the control of the lacUV5 promoter in pPSV37		This study
pP37-*acrHpcrH*-*popB*(S321C)-*popD*	Plasmid encoding *acrH*, *pcrH*, *popB*(S321C) and *popD* under the control of the lacUV5 promoter in pPSV37		This study
pP37-*acrHpcrH*-*popB*(S321C, 225VSVG)-*popD*	Plasmid encoding *acrH*, *pcrH*, *popB*(S321C, 225-VSVG) and *popD* under the control of the lacUV5 promoter in pPSV37. PopB has 4x VSV-G tag inserted after codon 225.		This study
pEXG2-*pcrV*(Q87C)	Allelic exchange vector designed to introduce Q87C mutation in *pcrV*.		Armentrout 2016
